# Psychiatrist experience of remote consultations by telephone in an outpatient psychiatric department during the COVID-19 pandemic

**DOI:** 10.1017/ipm.2020.51

**Published:** 2020-05-22

**Authors:** C. Olwill, D. Mc Nally, L. Douglas

**Affiliations:** Department of Old Age Psychiatry, Carew House, St. Vincent’s University Hospital, Dublin, Ireland

**Keywords:** COVID-19, Ireland, remote consultation, telemedicine, telephone consultation, telepsychiatry

## Abstract

**Objective:**

In response to the COVID-19 pandemic, there has been a shift globally from face-to-face consultations to remote consultations. In our department, remote consultations have taken in the form of telephone consultations. In this paper, we set out to study a group of Irish psychiatrists’ experience of these consultations.

**Methods:**

We identified recurrent themes in the existing literature on doctors’ experience of telephone consultations with a view to determining the applicability of these themes to a group of Irish psychiatrists. A questionnaire was developed based on themes in the literature. This was sent to all psychiatrists working in a busy psychiatric service in Dublin.

**Results:**

The questionnaire response rate was 72% (*n* = 26/35). Diagnostic challenges, the effect of phone consultation on the therapeutic alliance, challenges associated with the use of technology and ethical concerns were identified as issues. Flexibility in the working day and convenience were identified as possible benefits to telephone consultations.

**Conclusions:**

The group that participated in this research study identified a number of challenges to carrying out successful phone consultations. This study highlights the need at our clinical site for interventions to address the issues identified by staff. The findings also highlight the requirement for larger studies with stronger methodologies to determine the generalisability of our results.

## Introduction

In December 2019, an outbreak of a novel coronavirus disease was reported in Wuhan, China (Zheng, 2020). This novel coronavirus was later named COVID-19 (Jiang *et al.* 2020). The World Health Organisation declared the spread of COVID-19 to be a global pandemic on 11 March 2020 (World Health Organisation, 2020). In an attempt to reduce the opportunities for transmission of the disease, there has been an almost overnight change in the modus operandi of health services globally, with many of these changes likely to remain long after the current crisis has subsided (Thornton, 2020). One of these changes is a shift from traditional face-to-face consultations to remote consultations (Corruble, 2020; Greenhalgh *et al.* 2020; Wright & Caudill, 2020).

Telemedicine was first described in the 1970s and at that time was defined as the process of providing health care from a distance through the use of communications technology (World Health Organisation, 2010). Telepsychiatry is the field of telemedicine as applied to the speciality of psychiatry (American Psychiatric Association, 2019). Telephone consultations can be considered a rudimentary form of telemedicine (Downes *et al.* 2017). Since the time of its inception, telemedicine has been lauded by policy makers as a field with the power to transform healthcare provision in rural areas and in areas where services are underdeveloped (Bagchi, 2006). However, prior to the current pandemic, it had not achieved the widespread implementation that its proponents had hoped for. Lack of acceptance by clinicians is often cited as the most significant factor in its limited uptake (Cowan *et al.* 2019).

Telephone consultations can be described as ‘the process whereby patients receive medical advice by one or more qualified healthcare professionals via the telephone’ (Vaona *et al.* 2017). A 2018 review of the available literature on telephone consultations highlighted doctors mixed feelings with regard to telephone consultations (Van Galen & Car, 2018). This review found that while clinicians recognise the potential of phone consultations to improve access and choice for patients they have concerns about the effectiveness and safety of consultations given the lack of visual cues and lack of opportunity to physically examine the patient. A study looking at general practitioners’ experience of telephone consultations found that the lack of both of these aspects of the consultation affected GPs confidence in making a diagnosis (Foster *et al.* 1999). A recent mixed-methods study looking at the experiences of GP trainees undertaking telephone consultations echoed these findings stating that the main reasons for doctors reporting negative experiences of phone consultations were the absence of non-verbal cues and absence of the possibility of physically assessing the patient (Chaudhry *et al.* 2020).

Clinicians have previously cited increased flexibility and shorter consultation times as possible benefits to phone consultations as well as noting many possible benefits from a patient’s perspective including less waiting time, lowered costs, no need to travel and less commitment on their behalf (Car & Sheikh, 2003).

The therapeutic alliance is central to our practice as psychiatrists (Gaston, 1990). Any encroachment on the quality of this important factor will adversely affect patient care. The British Association for Counselling and Psychotherapy outlines competencies that clinicians should have in order to maintain the therapeutic alliance during telephone consultations – these include the ability to establish ground rules and boundaries, the ability to manage the impact of disinhibition, the ability to identify and manage risk and the ability to conclude the therapeutic relationship (British Association for Counselling and Psychotherapy, 2020). Boundaries are agreed limits in a psychotherapeutic relationship – they include consistency in terms of consultation start time, finish time, duration and also that the focus of the consultation remains on the patient (British Association for Counselling and Psychotherapy, 2018).

Doctors have expressed their concern about Medicolegal issues arising consequent to telephone consultations (Brant *et al.* 2016). Both the Irish Medical Council and Medical Protection Society have issued guidelines around carrying out remote consultations. These guidelines highlight the importance of documenting the reason for the remote consultation, obtaining informed consent from the patient, ensuring that the patient is adequately assessed and that patient confidentiality is maintained (Irish Medical Council, 2019; Medical Protection Society, 2020). A Cochrane review highlighted the limited training that doctors receive with regard to telephone consultations (Vaona *et al.* 2017).

It is worth noting that although the terms telemedicine and telepsychiatry were originally devised to include both established and novel forms of communications technology, for example, telephone, fax, email, Internet and videoconferencing, much of the research in telepsychiatry now uses the term telepsychiatry interchangeably with the term videoconferencing (Chakrabarti, 2015). The current study places a focus on telephone consultation as it is currently the primary method of communication with patients in our department.

In this paper, we set out to examine a group of psychiatrists’ experience of telephone consultations during the COVID-19 pandemic. We aimed to do this by identifying recurrent themes in the existing literature on doctors’ experience of telephone consultations. We then used a questionnaire to determine the applicability of these themes to a group of psychiatrists working in an outpatient psychiatric service in Dublin, Ireland.

## Method

Given the lack of consensus in the literature on the definition of telemedicine and telepsychiatry and in order to ensure relevance of our literature review to telephone consultations, our search terms focussed on telephone consultations. We initially searched PubMed and the Cochrane Library using search terms including ‘telephone consultation’, ‘psychiatry’ and ‘remote consultation’. This initial search had resulted in predominantly studies involving paediatric population which were not the target population of our own study. We subsequently widened our search terms to ‘telephone consultation’ and ‘medicine’ which returned articles predominantly focusing on general practice. Given the lack of available literature on adult psychiatric outpatient consultations, we felt that the articles found could be used as an evidence base for our questionnaire. We also used references of identified articles to find additional literature, and we reviewed Irish and international professional guidelines on the subject of telephone consultations.

We created a questionnaire that was informed by issues identified in our review of the literature on telephone consultations. Each question in the questionnaire was based on one of these issues. The questions asked in the questionnaire are shown in the appendix (Supplementary Material). Respondents were asked about their experience of diagnostic, technical, ethical and relationship issues during telephone consultations compared with face-to-face consultations. Respondents were also provided with a ‘free-text’ area to allow for additional comments. Demographic details including age and level of training were gathered. Non-Consultant Hospital Doctor (NCHD) training stages were divided into non-training posts, Basic Specialist Training (BST) and Higher Specialist Training (HST).

We chose a digital questionnaire due to its increased speed of data collection, the reduced requirement for face-to-face contact and its low-cost requirements. It also provided an opportunity to directly reference themes already explored in the literature. We adhered to the principles of good questionnaire design including piloting the questionnaire with a subgroup before wider dissemination and reducing the possibility of forced choice by including options in the questionnaire such as ‘unable to comment’.

The study protocol was approved by the ethics committee of the participating institution. We sent questionnaires to all medical staff within our department. These were NCHDs and consultants in a busy psychiatric service in Dublin. Consent was obtained from all participants. The request to complete the questionnaire was sent via an email containing a link to the online questionnaire. All those asked to complete the questionnaire were advised of the aim of our study. All responses were anonymised, and we advised all participants of this in advance.

Following data collection, the comments in the ‘free-text’ area were grouped into positive, neutral and negative comments by the lead researcher. Allocation to a given category was then confirmed by a second researcher. Common beliefs among the respondents were arrived at using word counting and pile sorting. The common themes were agreed and identified by consensus of the researchers (CO, DMN and LD).

## Results

The questionnaire response rate was 72% (*n* = 26/35). Age distribution and training-level distribution of the respondents are shown in Tables 1 and 2, respectively. Table 3 shows respondents’ level of agreement with each theme identified in the literature.

**Table 1.** Age distribution of questionnaire respondents. Total number of respondents, *n* = 26

Age	Number of respondents	Number as a percentage of total respondents (%)
25–34	10	38
35–44	9	35
45–54	2	8
55–64	4	15
65+	1	4

**Table 2.** Training-level distribution of questionnaire respondents. Total number of respondents, *n* = 26

Training level	Number of respondents	Number as a percentage of total respondents (%)
BST	5	19
HST	4	15
Non-training post	5	19
Consultant	12	46

### Diagnostic issues

Diagnostic challenges were the area in which our respondents almost universally agreed with the views found in the literature. Respondents were asked if their confidence in making a diagnosis was reduced, unchanged or increased during phone consultations. Ninety-two percent of the respondents (*n* = 24) agreed that their confidence in making a diagnosis was reduced. This reduction in confidence was most marked in the consultant group with 100% of consultants, (*n* = 12) noting a reduction in confidence making a diagnosis. Eighty-six percent of NCHDs agreed that their confidence in making a diagnosis was reduced. A large percentage of respondents (96%, *n* = 25) agreed that the lack of visual cues during phone consultations affected their assessment of the patient, this was most marked in BST with 100% (*n* = 5), agreeing that this was an issue. Ninety-two percent of consultants agreed that the lack of visual cues affected their assessment. Ninety-two percent of consultants agreed that risk assessment was more difficult over the phone than in face-to-face consultations. BST and HST trainees noted it to be an issue with 100% of these trainees agreeing with the statement. Seventy percent of respondents overall agreed that they found it more difficult to consider discharging a patient, this had a downward trend as respondents’ level of psychiatric training increased. Eighty percent of BST trainees agreed that it was more difficult to discharge a patient, whereas seventy-five percent of HST trainees and sixty-seven percent of consultants found this to be the case.

Thirty-nine percent of respondents agreed that they experienced a reduction in confidence in prescribing medication. This was most marked in the BST group with 60% of this group agreeing that their confidence in prescribing was reduced.

### Therapeutic alliance issues

Another commonly reported area of concern in the literature was the effect that phone consultations had on the therapeutic alliance. Eighty-eight percent of respondents agreed with the statement that they found it more difficult to establish an atmosphere of openness and trust with new patients on the telephone. This was least marked in respondents in the youngest age range of 25–34 with 70% (*n* = 7) describing it as an issue. In the group studied, visual cues affecting rapport was reported as an issue more often in those with lower levels of training (Table 3). Consultants were the group (67%) that agreed most frequently with the statement that it was more difficult than usual to establish roles and boundaries in a phone consultation, with only 21% of NCHDs trainees noting it as an issue.

**Table 3.** Respondents’ level of agreement with themes identified in the literature on doctors’ experience of telephone consultations relative to face-to-face consultations

Theme identified in the literature	Consultant (%)	NCHD (%)
Diagnostic		
Less confident in making a diagnosis	100	86
Lack of visual cues affected patient assessment	92	100
Risk assessment was more difficult	92	86
More difficult to consider discharging a patient	67	71
Did not feel that undergraduate education prepared me for phone consultations	92	86
Less confident prescribing medication	33	43
Therapeutic alliance		
More difficult to establish atmosphere of openness and trust with new patients	100	79
Patients disclosed sensitive information more freely	17	7
Roles and boundaries were more difficult to establish	67	21
Lack of visual cues affected ability to establish rapport	67	57
Ethical issues		
Medicolegal issues becoming more of a concern	42	64
Increased concern around patient confidentiality	33	29
Adequate level of supervision	42	71
Technical issues		
Technical issues such as the quality of the phone line were an issue	83	71
Assessing those who had issues with English language fluency was more difficult	86	75
Assessing those with cognitive impairment became more of an issue	100	100
Assessing those with hearing deficits became more of an issue	78	67
Practical issues		
Offered increased flexibility in the working day	83	86
Consultation times were shorter	67	79
Concluding interview was less difficult	25	57

The number of consultants who agree with each statement is shown as a percentage of the total number of consultant respondents (*n* = 12). Likewise, the number of NCHDs who agree with each statement is shown as a percentage of the total number of NCHD respondents (*n* = 14).

### Ethical issues

Forty-two percent of consultants agreed that they were more concerned about medical legal issues during phone consultation than during face-to-face consultations. This was higher for NCHDs with 64% of them agreeing that medicolegal issues became more of a concern for them during phone consultations. Thirty-three percent of consultants and twenty-nine percent of NCHDs’ reported increased concern about patient confidentiality.

### Practicalities

Respondents agreed with some of the possible time-saving aspects of phone consultations reported in the literature. Respondents agreed that phone consultations gave them more flexibility in their working day – 83% of consultants and 86% of NCHDs agreed with this statement. Respondents also agreed that phone consultations were shorter than face-to-face consultations – 67% of consultants and 79% of NCHDs agreed with this statement. Twenty-five percent of the total number of consultants who completed the questionnaire and fifty-seven percent of the total number of NCHDs who completed the questionnaire found it less difficult than usual to conclude a consultation.

### Technical issues

Technical issues were recognised as being an issue by both the consultant group and the NCHD group. Eighty-three percent of consultants agreed that technical issues such as the quality of the phone line was an issue, whereas 71% of NCHDs agreed that this was their experience. One hundred percent (*n* = 8) of those who attempted phone consultations with those with cognitive impairment found it to be more of an issue than it would have been in face-to-face consultations. Seventy-eight percent of consultants who attempted consultations with those with hearing impairments found it to be an increased issue, this was a less common finding in the NCHD group (Table 3). Eighty-six percent of consultants who attempted consultations with those who had a reduced fluency in English found it to be an increased issue, this was a less common finding in the NCHD group, 75% of whom noted it this to be the case.

### Analysis of themes from free-text question in the questionnaire

Sixty-five percent of the total respondents (*n* = 17) completed the free-text question asking respondents to describe their experiences on phone consultations. The responses were grouped into three categories depending on the respondents’ attitude to phone consultations. Four respondents displayed a positive attitude, four displayed a neutral attitude and nine displayed a negative attitude.

Examples of positive and negative responses are shown in Table 4.

**Table 4.** Examples of positive and negative responses given in the free-text question in the questionnaire

Positive responses	Negative responses
‘More time efficient’	‘I don’t trust that I am getting anything like the same sense of the other person that I get when face to face and that has to impact everything’
‘Added flexibility’	
‘More convenient’	‘It felt more like a check-in, than a true appointment/review of mental state’
‘I do think they could have a place after COVID, in well selected patients’	‘Phone consultations cannot replace face to face consultations’

Respondents were asked to type any thoughts on their own experiences of telephone consultations and how they compare with face-to-face consultations. These were then grouped into positive, negative and neutral comments by consensus of the researchers.

Using word counting and pile sorting, the researchers had consensus that there were two recurrent beliefs based on the ‘free-text’ question:

(1) the belief among the respondents that telephone consultations cannot replace face-to-face consultations and
(2) the belief among the respondents that telephone consultations are only suitable for certain subsets of patients.

## Discussion

Phone consultations have been incorporated into our psychiatric service at unprecedented speed. This study reports on the experiences of a group of psychiatrists in a busy outpatient psychiatry department. The study findings indicate that the study group largely agrees with the experience of doctors in other specialties undertaking telephone consultations as described in the previous literature.

Diagnostic challenges were the area in which our respondents almost universally agreed with the views found in the literature. Our group of psychiatrists agreed with the literature findings which found that doctors’ confidence in making a diagnosis was reduced during telephone consultations (Foster *et al.* 1999). Interestingly in the group studied, this reduction in confidence was most marked in the consultant group with 100% of consultants (*n* = 12), asserting that their confidence in making a diagnosis was reduced in phone consultations. The respondents’ confidence in prescribing medication was not correspondingly reduced (33% of consultants and 43% of NCHDs had reduced confidence in prescribing). One would expect that if clinicians find diagnosis difficult that confidence in prescribing would be correspondently reduced. This requires further exploration in future research.

Our respondents agreed that the Therapeutic Alliance was affected by the consultation taking place over the phone as opposed to in person. The importance of meeting a patient face to face in the establishment of a therapeutic alliance is highlighted here and underlines the need for physical and visual relating as well as verbal communication in the establishment of a meaningful trust and rapport. Difficulties in establishing rapport and trust coupled with difficulties with boundaries obviously are a concern. This could affect the accuracy of assessments, the effectiveness of any interventions and levels of satisfaction for clinicians whilst doing their assessments. The importance of establishing these aspects of therapeutic alliance during phone consultations is highlighted in the guidelines by the British Association for Counselling and Psychotherapy (British Association for Counselling and Psychotherapy, 2020). Of note, the younger the respondent the less likely they were to experience difficulty in establishing rapport. This may be due to generational issues such as younger generations’ immersion in social media as a means of socialising. However, this requires further exploration. Unusually, NCHDs experienced less difficulties than consultants when setting rules and boundaries via telephone consultations. A further exploration of this surprise finding would help understand if this is because NCHDs generally are required to set fewer boundaries or perhaps are seeing a patient cohort who is less challenging to manage.

Interestingly, medico legal issues highlighted in the literature (Brant *et al.* 2016) were not a universal concern among our group with only 42% of consultants but 64% of NCHDs noting it to be an increased concern during phone consultations. This is surprising given clinicians usual attention to medico legal issues and requires further exploration.

In agreement with a recent Cochrane review (Vaona *et al.* 2017), our respondents endorsed that their undergraduate training did not prepare them for phone consultations, which has implications for future research looking at doctors’ training.

One of the common beliefs shared in responses to the ‘free text’ section was that phone consultations could not replace face-to-face consultations. This is an interesting attitudinal finding when considered in the context of the finding that clinicians are often ‘the gatekeepers’ (Cowan *et al.* 2019) to the implementation of telepsychiatry.

Our group of clinicians’ positive experiences of phone consultations centred around the themes of increased flexibility and convenience in the working day – mirroring that of the published research (Car & Sheikh, 2003).

Two interesting issues emerged in our study that are relevant to service provision in the current COVID-19 pandemic. The first was that 100% of those respondents who carried out a phone consultation with a patient with cognitive impairment found that their assessment was more difficult than a face-to-face consultation. As Ireland currently operates a policy where it is compulsory for those over 70 to stay home (Cocooning), this makes the provision of effective, safe psychiatric care more challenging for those over 70 with cognitive impairment. It is likely that structured neurocognitive tests designed for use over the telephone and, in time, video consultations could go some way towards mitigating this issue. The second issue is that 70% of doctors found it more difficult to consider discharging a patient on the phone. This has obvious implications for psychiatric services resources going forward.

Strengths of this piece of research is the high response rate that we had to the questionnaire – 72% (*n* = 26/35). It is as far as we know it the first piece of research to look at Irish psychiatrists’ experience of phone consultations during COVID-19 making it a contemporaneous and timely study.

Weaknesses of the study include the small sample size and that the questionnaire was only sent to doctors at one hospital site. We did not record data on the respondents’ frequency of telephone consultations or how much of their work load was over the telephone as opposed to face to face. It is also worth noting that we measured a subjective phenomenon, that is, doctors’ opinions on the topic of remote consultations as opposed to making an objective measure of the effectiveness of phone consultations relative to face-to-face consultations. The review of the literature which informed the development of the questionnaire was not an extensive or systematic review of the literature. We also recognise the limitations of using a questionnaire including its possible introduction of bias into the research including social desirability bias. This is a type of response bias in which survey respondents have a tendency to answer questions in a manner that will be viewed favourably by others. Recall bias is also incorporated into our questionnaire as it asked respondents to reflect on their past experiences. One further limitation of our questionnaire is that, although we piloted the questionnaire with a subgroup before wider dissemination, it was not validated.

Future studies could carry out a more comprehensive literature search to inform the development of the questionnaire and the questionnaire could be disseminated to multiple clinical sites. It is of course imperative that any refinement of how doctors and patients engage into the future is informed by further study of the opinions of both doctors and patients.

## Conclusion

Our study extends the existing evidence base by looking at Irish psychiatrists’ experience of telephone consultations. The group studied agreed with many of the themes already found in the literature on telephone consultations. The finding that many of the psychiatrists noted an increased difficulty carrying out assessments and decreased confidence in diagnosis has implications for service development at our clinical site. An example of such an intervention might be a dedicated teaching on the topic of telephone consultations and telepsychaitry informed by the literature and this study. Larger studies with stronger methodologies that encompass multiple clinical sites are required to investigate the generalisability of our findings. Our study highlights the requirement for ongoing research in this area in order to make the change to phone consultations safer, more easily adapted and sustainable.

As more advanced forms of technology such as videoconferencing software become available to the irish health service, future research will be needed to determine if issues identified with remote consultations by telephone remain an issue or if new issues arise. For now, it is likely that the innovative use of a combination of face-to-face and telephone consultation may be necessary to reduce the risk of the spread of COVID-19, whilst providing optimal patient care and assessment.

## Supplementary material

For supplementary material accompanying this paper visit http://dx.doi.org/10.1017/ipm.2020.51. click here to view supplementary material
