# Supplementary material for: Psychiatrist experience of remote consultations by telephone in an outpatient psychiatric department during the COVID-19 pandemic
Source: Ir J Psychol Med. 2020 May 22:1–8. doi: 10.1017/ipm.2020.51 (PMC7283108; doi:10.1017/ipm.2020.51)
Supplement: Supplementary file 1 [file S0790966720000518sup001.docx]

| **Doctors’ Experiences of Phone Consultations During the Current Covid -19 Pandemic** |
| --- |

In completing this questionnaire we would ask that you reflect on your recent phone consultations and how they compare to your usual face to face consultations. Each statement in the questionnaire is followed by 2-3 possible responses. Please read each statement carefully and select the response that best captures your experience. There is a space near the end of the survey for you to free type any thoughts on own experiences of telephone consultations.  Thank you!

Reponses given in this questionnaire are fully anonymous

Top of Form

1. I felt my undergraduate training prepared me for phone consultations

Yes

No

2. When carrying out a consultation over the phone as opposed to in person my confidence in making a diagnosis was

Reduced

Unchanged

Increased

3. When carrying out a consultation over the phone as opposed to in person my level of concern about medicolegal issues was

Reduced

Unchanged

Increased

4. There have been incidences were technical issues such as the quality of the telephone line were an issue for me

Yes

No

5. I felt phone consultation awarded me flexibility in the working day that I did not have with face to face consultations

Yes

No

6. When carrying out a consultation over the phone as opposed to in person my confidence in prescribing medication was

Reduced

Unchanged

Increased

7. When carrying out a consultation over the phone as opposed to in person issues around patient confidentiality were

Reduced

Unchanged

Increased

8. When carrying out a consultation over the phone as opposed to in person risk assessment was w

More difficult

Unchanged

Less difficult

9. Did cognitive impairment become more of a barrier to communication during phone consultations?

Yes

No

Unable to comment

10. Did hearing deficits become more of a barrier to communication during phone consultations? w

Yes

No

Unable to comment

11. When carrying out a consultation over the phone establishing roles and boundaries in a consultations was

More difficult

Unchanged

Less difficult

12. Did patients lack of fluency in English language become more of a barrier to communication during phone consultations w

Yes

No

Unable to comment

13. My consultation times were

Shorter

Unchanged

Longer

14. The lack of visual cues affected my ability to assess the patient

Yes

No

15. The lack of visual cues affected my ability to establish rapport with the patient

Yes

No

16. Concluding interviews was

More difficult

Less difficult

Unchanged

17. For cases which I was considering discharging I found the decision to discharge

More difficult

Less difficult

Unchanged

18. I felt I had an adequate supervision framework for doing phone consultations

Yes

No

19. Did you find that patients disclosed sensitive information

More freely

Less freely

An unchanged amount

20. With new patients, establishing an atmosphere of openness and trust over the telephone was: w

Easier than usual

More difficult than usual

No different than usual

21. Please type any thoughts on own experiences of telephone consultations and how they compare with face to face consultations.

22. My level of training is

BST1/BST2/BST3 HST Consultant

                                                                                                            

23. Age

25-34,35-44,45-54,55-64, 65+

                                                                                                                                     
